# Mental Health and Integration: A Qualitative Study on the Struggles of Recently Arrived Refugees in Germany

**DOI:** 10.3389/fpubh.2021.576481

**Published:** 2021-11-04

**Authors:** Lena Walther, Diana Rayes, Julia Amann, Uwe Flick, Thi Minh Tam Ta, Eric Hahn, Malek Bajbouj

**Affiliations:** ^1^Department of Psychiatry and Psychotherapy, Charité University Medicine Berlin, Berlin, Germany; ^2^Department of Education and Psychology, Freie Universität Berlin, Berlin, Germany

**Keywords:** refugees, asylum seekers, migration, mental health, well-being, integration, qualitative study

## Abstract

**Introduction:** Forcibly displaced people are at particular risk of mental health problems and also face specific integration challenges upon resettlement. Existing literature suggests that there may be a bidirectional relationship between mental health and integration. The present study seeks to understand the relationship between integration processes and mental health problems or significant negative emotional experiences among adult refugees in Germany.

**Method:** Applying a qualitative approach, we conducted 54 semi-structured interviews with refugees and asylum seekers who arrived in Germany between 2013 and 2018 currently residing in Berlin, Leipzig, or the Duisburg area in North Rhine-Westphalia. Data was collected between December 2018 and September 2019. We analyzed transcripts inductively using thematic analysis.

**Results:** Five themes covering the various links between integration and mental health problems or significant negative emotional experiences were identified. First, we found that the mental health consequences of past adverse experiences, as well as ongoing worries about those left behind in the homeland, can seriously impede refugees' ability to pursue activities key to integration. Second, the process of applying for and securing asylum can result in uncertainty and fear, which, in turn, burden the individual and may impact motivation for integration. Third, many of our participants described mental health ramifications related to feeling stuck and thwarted in the pursuit of building a life, especially in securing employment. Fourth, some participants described feeling so overwhelmed by fundamental tasks throughout the integration process, namely, language learning and bureaucratic processes, that these take a psychological toll. Fifth, we identified several forms of social disconnection between refugees and members of the host community due to xenophobia, social and cultural differences, physical and emotional isolation in refugee camps, as well as with co-nationals and fellow refugees. Negative emotions, mistrust, and socio-cultural differences that emerge throughout the integration processes seem to erode social cohesion among refugee communities, potentially further threatening mental health.

**Conclusion:** Mental health problems and integration processes appear to be closely related across different areas of integration. Innovative solutions to challenges identified by members of the refugee community in Germany stand to benefit mental health and integration outcomes simultaneously.

## Introduction

The integration of refugee and asylum-seeking populations (henceforth referred to as “refugees”) is a pressing challenge for host societies worldwide. While the concept of integration has been extensively debated in the literature (1–3), it can be understood to represent a “process of becoming an accepted part of society” [(4), p. 11]. This process is multidimensional, encompassing legal, political, economic, social, and cultural dimensions (4, 5). It is also increasingly seen as a bidirectional process involving both migrant and host society communities (6). For refugees, integration can be particularly challenging (7) because, unlike other migrants, refugees are often unable to plan their futures or choose their destinations. They also face several structural barriers in the host country context, including uncertainty regarding residency status and discrimination.

Within their prominent framework for refugee integration, Ager and Strang (2) position health as one of the “markers and means” of integration (p. 170). More generally, it has been posited that health, including mental health, is a prerequisite for integration as well as a positive outcome of successful integration trajectories [e.g., (8)]. Refugees who resettled in Western countries have repeatedly been found to be at particular risk of poor mental health (9–12), showing high prevalences of symptoms of depression, anxiety disorders, and PTSD (13–16). While past research has focused on mental health problems as a consequence of traumatic experiences in pre-migration contexts or during migration [e.g., (17, 18)], it is now recognized that post-migration living conditions, including the circumstances and processes of integration, are also linked to mental health outcomes [e.g., (19)].

Previous, mostly quantitative research has identified a range of associations between various mental health measures and different aspects of integration. These include legal matters such as status insecurity (20), challenges around family separation and reunification (21, 22), socio-economic stressors such as poor housing conditions (23), unemployment [e.g., (24)], as well as social and interpersonal stressors such as host country language learning (25) and discrimination experiences (26). While most research has emphasized the impact of post-migration conditions on mental health (19), fewer explicitly consider the opposite effect: the effect of poor mental health on the processes of integration (27–29).

Qualitative approaches, which have been under-utilized in the study of refugees' experiences (30, 31), are ideally suited to delving deeper into the nuances of potential associations between integration and mental health. They are better suited than quantitative approaches for illuminating mechanisms behind these associations, providing insights at a level of specificity that is close to lived experience, and identifying previously unknown challenges by allowing individuals from the community under study to give their personal accounts (30–33). In mental health research, qualitative studies can contribute to a more specific understanding of what individuals are experiencing than standardized symptom checklists (33), which is particularly valuable in a cross-cultural research setting. Existing qualitative studies on refugees' experiences regarding the relationship between mental health and integration have explored specific domains of integration, such as housing (34) and social integration (35), full ranges of stressors and mental health ramifications (31, 36), and overall integration experiences with insights on well-being (37, 38). However, we have not come across qualitative analyses focused explicitly on the link between integration processes and mental health struggles. Our study addresses this relationship in a diverse sample of refugees within their first 5 years in Germany.

Germany is among the five countries globally that have received the largest number of refugees, with ~1.1 million living within its borders in 2018 (39). Just over half of refugees who arrived in Germany at the height of the influx were of Syrian (41.5%) or Afghan (9.8%) nationality (40). With 890,000 new registrations by refugees in Germany in 2015 alone (41), refugee integration became a focus of political and public discourse (42, 43) against a backdrop of anti-refugee sentiments on the rise (43). One cornerstone of integration policy in Germany is the asylum procedure, which can take several years including appeals due to a large number of asylum applications. There are several different types of residence status, with varying durations and levels of access to institutions (44). Providing housing has also proved a challenge: for up to 6 months, new arrivals are placed in refugee camps; then, municipal governments are responsible for providing housing, usually in shared refugee housing facilities (45). Finally, efforts have been made to ease major aspects of structural integration, namely integration into the workforce or education programs, with first steps taken to tackle bureaucratic obstacles like certificate recognition and legal restrictions (46). “Integration courses,” which provide language and civics instruction, are essential in this process; however, access is not guaranteed due to high demand (47).

The present study offers detailed insights into the relationship between integration and mental health struggles among refugees who arrived in Germany during the peak influx years between 2013 and 2018 and resided in one of three urban areas—Berlin, Leipzig, or greater Duisburg—at the time of the interviews. Various aspects of building a new life in Germany and a range of mental health problems or significant negative emotional experiences reported by participants were examined. We aim to provide insights to integration and health policymakers, health care providers, and researchers, particularly in light of recent changes in asylum-seeking and integration policies in the German context.

## Materials and Methods

### Participants and Sampling

The study sample included 54 participants living in urban parts of Germany, including Berlin, Berlin; Leipzig, Saxony; Mülheim an der Ruhr, Dinslaken, or Duisburg, North Rhine-Westphalia, Germany (see Table 1 for sample characteristics). These locations were chosen to capture experiences from cities of different sizes from former East and West Germany. Recruitment from rural areas was not attempted due to logistical constraints, including the need for in-person access for interviewers and the limited size of the refugee population in rural areas. Participants were recruited through community outreach via social media and refugee organizations (see Appendix for advertisement) and snowball sampling. Our single inclusion criterion was having arrived in Germany in 2013 or later through forced migration (self-identified). We became increasingly selective in our recruitment to pursue the goal of maximum variation sampling (48) by age, gender, educational background, and country of origin. We continued recruitment until these sampling goals were achieved. Of the people initially interested in participating, 31 either withdrew or were excluded due to not meeting the inclusion criterion.

**Table 1:** Sample characteristics.

Gender	Female	Male					
	24	30					
Age	18–24	25–29	30–34	35–39	40–44	45–49	50–55
	11	13	12	5	5	3	5
Country of origin	Syria	Afghanistan; Afghanistan/Iran	Iran	Pakistan	Palestine	Libya	Sudan
	36	9	4	2	1	1	1
Level of education	No secondary education	Secondary education	Started university in country of origin	University-educated	Young and currently in secondary education	N.A.	
	5	3	9	28	3	6	
Residence in Germany	Berlin, Berlin	Leipzig, Saxony	Mülheim an der Ruhr, Duisburg, or Dinslaken, North Rhine-Westphalia				
	39	4	11				
Year of arrival in Germany	2013	2014	2015	2016	2017	2018	
	1	1	34	11	6	1	
Legal status	Refugee or asylum status	Subsidiary protection or deportation ban	Unresolved	Humanitarian program	Family reunification	Visa sponsorship	N.A.
	25	10	11	1	3	1	3
Housing	Private	Housing facility	N.A.				
	30	15	9				
Occupation	Gainfully employed	In education	None				
	13	11	30				

In the Results below, participants' ages are given within a 5-year range with each quote, gender is given in capital letters “F” or “M” behind the age (e.g., “30–35F”). When the country of origin or another attribute is important to name, participants' genders are obscured, both in the text (“they”) and in the codes behind quotes (e.g., “age 30–35”) to protect their identities.

### Topic Guide

The topic guide (see Appendix) comprised a brief introductory section on migration history and living situation, and two main parts: one on cultural experiences, the second, which is the focus of this work, on mental health and emotional experiences. The topic guide has a broader scope than the present study because the interview study as a whole was part of a larger collaboration project. The present study is one of several investigations based on this data [see (49) on resilience among refugees]. We used general terms (“feelings”; “well-being”) to discuss mental health with participants to prevent stigmatization or hesitation in sharing personal thoughts. We followed general recommendations for constructing topic guides for semi-structured interviews [e.g., (50, 51)] and consulted community members regarding the best terminologies to describe mental health and well-being. A partial, preliminary version of the topic guide was piloted in eight interviews not included in this study. Some additional questions were added to the topic guide after the first few interviews.

### Data Collection

The interviews were conducted between December 2018 and September 2019 in Berlin, Leipzig, and Mülheim an der Ruhr, Germany. Semi-structured in-depth interviews were conducted face-to-face and mostly with individual participants. In a few cases, partners or translators were present (one interview of two brothers was analyzed as two separate interviews because both answered the questions). All interviews, except one, were audio recorded. Interviews took place at locations of participants' choosing (mostly quiet locations within public cafés).

Seven different interviewers conducted the study: a female Arabic-speaker (DR, Master of Public Health), a male Arabic-speaker with refugee status (sociologist in MA program), a male Farsi-speaker (professional translator in psychiatry setting), and four German- and English-speaking interviewers (psychologists and sociologists, MA, MSc, or BSc, including LW and JA). We instructed interviewers on the main study goals, interviewing practices, and the ethical framework.

Interviews were conducted in Arabic (23), Farsi/Dari (10), German (19), or English (3). Participants were able to choose the interview language and were matched with interviewers accordingly. Gender or other features were not taken into account in matching interviewees and interviewers; however, interviewees were aware of the gender of their interviewer before the interview. In the Duisburg area, the presence of a translator was required for some interviews because a German- and English-speaker conducted all of the interviews there. Beverages were purchased for participants, but there was no financial compensation. Most interviews took between 45 and 60 min; the shortest interview took ~30 min, the longest was around 90 min. The audio recordings were transcribed and translated into English or German by either the interviewers or external professional translators. To ensure the quality of the transcripts, we commissioned professional translators without previous study involvement to check a randomly selected interview by each transcriber.

### Data Analysis

We applied thematic analysis following Braun and Clarke's (52) guidelines to identify and organize patterns in our data. For data immersion, LW and JA read through all the transcripts. Next, we coded transcripts line-by-line for the second part of the interview and data segments that were pertinent to our primary research question in the rest of the transcripts. In the interest of validity, LW and JA independently coded half of the transcripts each, exchanging transcripts after about every five, discussing and amending codes. The data analysis was inductive for the most part. It was only deductive in the sense that we approached the data with the overarching research question “how do integration processes and mental health relate?” in mind.

Our codes were open and detailed enough to represent the meaning of the text segment accurately. After the initial coding of each transcript, we entered information on each participant into a table, including sociodemographic and migration background information, a summary of the interview, as well as a mental health status summary. The mental health status summaries included explicit mentions of mental health problems or treatment history by participants, as well as an assessment of overall functioning and well-being based on the interview content as a whole. Their purpose was to ensure that individual interview excerpts would not be taken out of context in the analysis. For example, a statement about a stressor by an individual whose interview reflected very high levels of well-being overall was not read as equally indicative of an integration process negatively relating to mental health as a similar statement by a struggling individual.

After coding all interviews, LW identified candidate themes revolving around the central research question by going through codes systematically and entering them into evolving theme maps in MAXQDA's visualization toolkit with feedback from JA. We continuously referred to the transcripts and our summary table to ensure including an across-case and a within-case view of context (53). While the results only include a few examples of a within-case perspective, individual interview passages were interpreted with regard to the case as a whole. Collaborators from the community under study critically assessed the themes for plausibility, and we refined them until a final thematic map (Image 1) was agreed upon.

### Identification of Mental Health Problems

All explicit mentions of mental health conditions or symptoms, such as “psychological problems,” depression, anxiety, trauma or post-traumatic stress, nightmares, or trouble sleeping, were regarded as indicative of mental health problems pertinent to our analysis, as were statements by participants expressing significant negative emotional states or experiences, including expressions of deep or lasting sadness, worries or rumination, exhaustion or listlessness, apathy, anger, fear, frustration, hopelessness, emptiness, overwhelm, loss of self-esteem or self-worth, loss of motivation, social withdrawal. In other words, our study explored the whole spectrum of poor mental health, not just the clinically significant end (54). This broad view is necessitated by the study design, which relies on participants' subjective expressions of experiences. We also attended to mechanisms that could cause or exacerbate mental health problems and make these instances explicit in the analysis.

### Concept of Integration

We considered all processes involved in arriving and building a new life in Germany mentioned by our participants as integration processes in our analysis, including, for example, legal processes, the housing journey, learning languages, navigating everyday life, interactions with cultural differences, pursuing education, employment, and other activities for oneself and others in the family, social life, experiences of interactions with key administrative institutions, feelings of inclusion and belonging, developments in one's sense of identity.

### Reflexivity

The study team included researchers of different ages, genders, levels of seniority, cultural backgrounds, as well as disciplinary backgrounds, including psychology, psychiatry, sociology, and anthropology. Furthermore, we consulted members of the community under study in the thematic analysis process: preliminary results were presented at a workshop and critically discussed.

## Results

**Figure 1:**
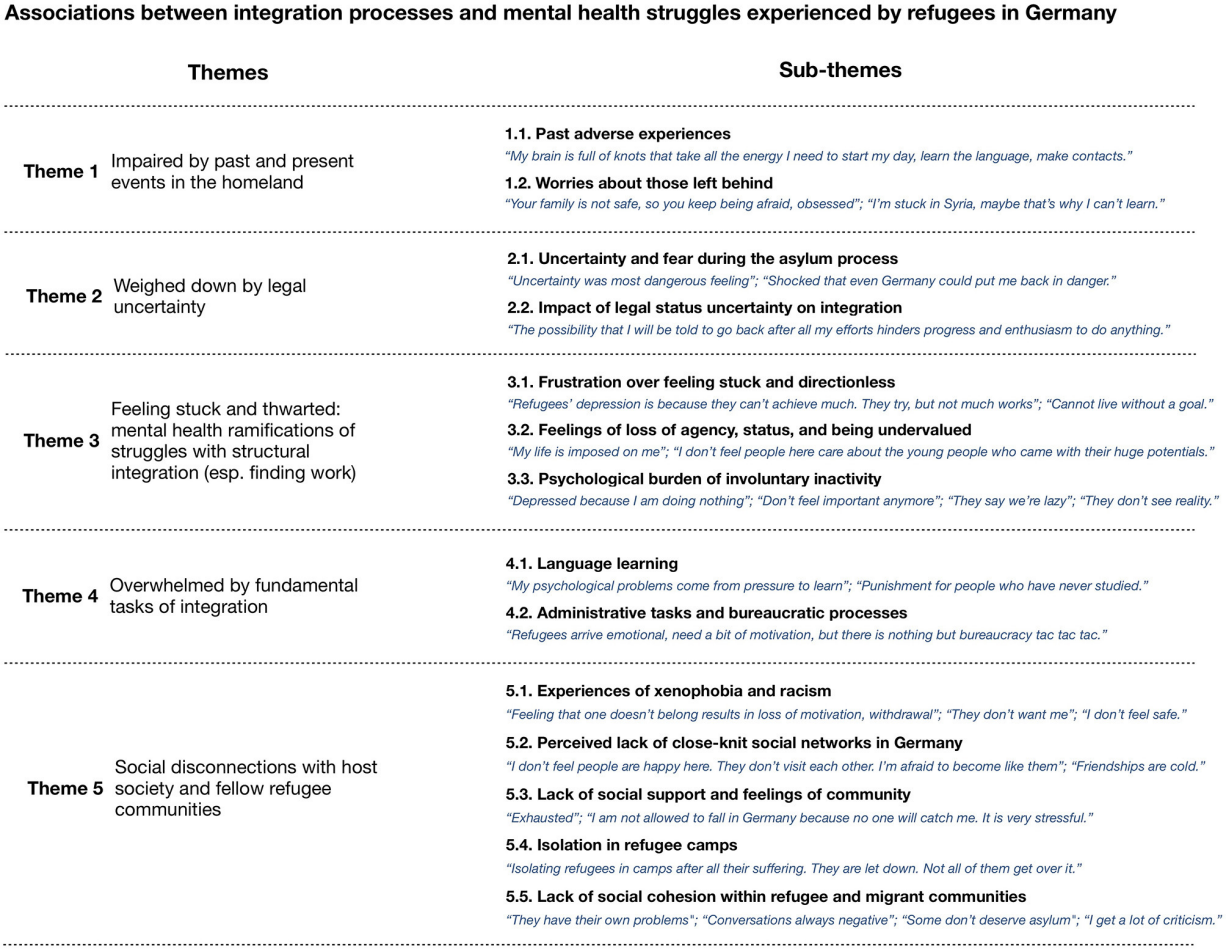
Thematic map. Quote segments in italics are abridged and partly slightly reorganized for brevity; semi-colons separate utterances by different participants.

We identified five themes, each with sub-themes (Figure 1). Theme 1 is clearly about poor mental health impacting integration unidirectionally. Themes 2 and 5 are mixed. Themes 3 and 4 are predominantly about the impact of integration processes on mental health, but they also capture several concomitant negative repercussions for integration and integration struggles exacerbated by mental health problems or negative emotional states. As the results below show, illustrating the relationship between mental health and integration separately for different areas of integration ended up being a central organizing principle in the thematic analysis due to the specific mental health and integration connections we found within each.

Each theme is described using quotations from the interview transcripts. The most illustrative expressions of an experience or multiple expressions that capture different facets of an experience were selected in order to reduce redundancy and provide a rich picture of the data.

### Theme 1: Impaired by Past and Present Events in the Homeland

This theme addresses how mental health problems linked to pre-migration adverse experiences (sub-theme 1.1.) and worries about the homeland and family members left behind (1.2.) can result in overall functional impairment and impede activities key to integration.

#### 1.1 Past Adverse Experiences

Several participants described feeling unable to tackle various aspects of their integration process because of poor mental health attributed to adverse or traumatic experiences before and during flight. For example, one participant (50-55F) attributed her language learning difficulties to past experiences: “I doubt I will ever learn this language. It is too hard for me because I have psychological problems. I've experienced so many problems in life. I can't just forget them. My brain doesn't have the capacity to learn so much at once.”

Another participant (30–34 M) who experienced traumatic events throughout his life in his country of origin reported feeling unable to “try to integrate” into German society “with a clear mind” until he has received therapy. He reported suffering daily from suicidal thoughts and nightmares concerning his experiences prior to arrival in Germany: “My brain is psychologically full of these knots. […] like in a vicious cycle, these [psychological problems] take all the energy I need to start my day and stay on my shoulders like a heavy weight. […] I still feel the pain even though I'm in Germany. This feeling takes all the energy I need for learning the language and for making social contacts.”

In a statement generalizing these deleterious effects of past adverse experiences, a Syrian participant (age 25–29) said that they have “noticed […] the Syrians […] have a kind of general depression, even if they don't want to admit it” from the war. They reported that this depression makes them “[lose] the ability to do anything” as soon as they encounter obstacles in their attempt to have a purposeful day. These sentiments were echoed by another Syrian participant (age 45–49) who says of themselves and their fellow Syrians, “We are devastated […], 7 years of war—we are psychologically destroyed.” They, too, feel they were “made unable to do things” by “the horror that [they] experienced in Syria and the fear of losing [their] children, the things [they] saw.”

#### 1.2 Worries About Those Left Behind

Some participants also feel impaired by concerns about family members still living in their country of origin: “My mom and my brother are still in Syria, so my head is full! I have no activities” (age 45–49). These worries have the potential to prevent refugees from overcoming their pasts and give rise to guilt and rumination: “When you come to Germany alone, you are safe then, but your family is not! So, you keep on being afraid, the same worries! […] because I came here, and I am in exile and left them, it became like an obsession to me [to check on them]” (25–29 M).

Intense and debilitating worries about the homeland and fellow citizens left behind, not just family members, were also reported by some participants. Continuous checking of the news is common among these participants, making them feel as if they are not rooted in their present circumstances and isolated from those around them, even co-nationals: “I am not out of Syria yet […], I'm still stuck there, and I use the internet in the morning to check the Syrian news, I listen to the radio about what is happening in Syria, and this is what my Facebook is all about, too. I can't forget and just start here […]. Maybe that's why I'm late learning the language, or that's why I can't remember words that I learn. […] Everyone around me asks me to get out of this grief, but […] I don't understand these people, sometimes I think they were not in the same war […]” (age 50–55).

### Theme 2: Weighed Down by Legal Status Uncertainty

The asylum process is central to refugees' post-migration experience since it determines access to various institutions, freedoms, and the right to stay in Germany. This central legal process of integration is bound up with distress due to uncertainty and fear (sub-theme 2.1.), and the burden of uncertainty has the potential to impact other areas of integration (sub-theme 2.2.) negatively.

#### 2.1 Uncertainty and Fear During the Asylum Process

Many of our participants reflected on the asylum process as a major stressor. Some participants expressed lasting distress over what happened during their asylum hearing, including regrets over what they said and inadequate translation in the hearings. However, the most significant mental health impact of seeking asylum among our participants is the burden of uncertainty that accompanies the process: “Until [you have an answer], you will always have fear. Always, […] It was uncertainty in my life that I considered the most dangerous feeling in my life. […] Since we had this positive answer [regarding our asylum application], yes, I am very happy, I don't take my medicines anymore” (25–29F). The duration of this phase of uncertainty and the relentlessness of the accompanying stress was emphasized. According to one participant (35–39F), “all people are psychologically tired” from hearing a succession of updates about their status “for a year, 2 or 3 years” - even after having been granted a title, waiting on the decision regarding its renewal.

The perceived lack of influence over the outcome of the asylum process, which can be understood as a loss of agency, was also highlighted as particularly challenging to deal with: “This burdens me immensely—not being able to do anything [to impact the asylum process] and just waiting to see what will happen” (25–29 F).

One participant (30–34 M) exhibited a severe fear of deportation. He reported not having been granted the complete protection status that he was expecting to receive. He “no longer feel[s] safe” and is shocked that “even Germany could put me back in danger.” His fear of deportation was exacerbated by previous traumas from political persecution and violent conflict, losing friends and relatives: “All my days are getting the same pattern where I get nightmares about [country of origin], or fears that I will get deported. […] Constant fear, anxiety, and nightmares.”

#### 2.2 Impact of Legal Status Uncertainty on Integration

The adverse mental health consequences of the uncertainty surrounding legal status can reduce the ability to perform tasks essential to integration. A participant (30–34 M) with severe fear of deportation said that “the stress takes everything out of” him and “doesn't give him the chance to feel that [he] wants to do any activities.” Another participant (18–24 M) reported experiencing symptoms of forgetfulness as a consequence of 4 years of uncertainty regarding his legal status that were “psychologically taxing.”

Furthermore, several participants described the lingering uncertainty itself as lowering their motivation to build a life in Germany: “The possibility that I will be told to go back after all my efforts here hinders progress, achievement and enthusiasm to do anything” (30–34 F). This quote also demonstrates how the possibility of being sent back can make refugees feel undervalued in their contributions, and “after all this effort […] feel still not accepted” because of the impression that Germany is “thinking about how to send us away” (18–24 F), threatening the sense of belonging to German society.

### Theme 3: Feeling Stuck and Thwarted: Mental Health Ramifications of Struggles With Structural Integration

A large cluster of stressors and associated psychological problems centers around a perceived struggle with structural integration, which here is short for: participation in the host country labor market and educational programs, facilitated by language and integration courses (although in some integration frameworks, it also includes legal dimensions and housing [see e.g., (55)]). Many of our participants expressed feeling unable to accomplish various aspects of structural integration. Restrictions on access to language courses and work permits due to legal status were frequently mentioned obstacles. Problems with the acceptance of certificates or otherwise seeing no future for one's profession in Germany as well as a perceived lack of guidance or too many restrictions (e.g., from the employment agency, “Job Center”) were also frequently mentioned, as were concerns about slow progress in language skill acquisition. Finally, several participants reported feeling held back by housing conditions impeding their pursuit of structural integration goals [e.g. “I wanted to study, but without an apartment and without privacy, I had to cancel everything. […] If I can't even sleep in peace, how am I supposed to work?” (18–24M); “I got the B1 certificate […] despite the conditions in the housing facility, which I cannot describe” (35–39F)].

The challenges of structural integration were related to a myriad of mental health consequences by our participants. Many attributed feelings of depression to feeling stuck and without direction (sub-theme 3.1.), a loss of agency, status, and a sense of being valued (3.2.) and felt burdened by involuntary inactivity because of slow structural integration (3.3.). This theme captures how integration processes impact mental health, and participant perspectives quoted in this section also strongly suggest the potential for vicious cycles wherein frustrations demotivate and make integration even harder in turn.

#### 3.1 Frustration Over Feeling Stuck and Directionless

Some participants attributed depression among refugees to frustrations over feeling unable to start a life in Germany, more so even than to past traumatic experiences: “[The other refugees] are suffering a little bit from depression. […] Not because of the war. It emerged here. Because of the difficulties, they can't achieve much […]. They try, but not much works out [pause] that's why” (30–34M). Indeed, several participants expressed feeling they have “accomplished nothing” (30–34F) or are “not developing” (18–24M) in their years in Germany, highlighting that career ambitions do not recede into the background in the flight context, especially among those of prime working age. Several participants in their thirties reported feeling under pressure to build a new career quickly. One participant (30–34F) considers “morning depression […] a must” because she is “already” in her thirties and has “no career […] because [her] university degree is irrelevant here.”

The lack of direction that comes from an inability to build an active life can itself represent a source of pressure. One participant felt that she struggles because she “cannot live” without a goal, that pursuing challenges “is life” (45–49F). Another participant (age 40–44), who reported seeing no future for her job in Germany, suffers from attempting to pursue goals while feeling a lack of direction: “I'm not pleased with my life here. I don't have a plan. I don't know what the plan for tomorrow is. Keeping going without destination, plus my other problems, makes me feel so tired.”

The burden of having no goals to pursue has troubled some refugees for many years, even the entire period of their displacement: “I've been suffering for the last seven years for not knowing where to go, what to do, […] I had to visit a therapist” (35–39M). As this quote shows, aimlessness from losing an established career might be compounded by uncertainty about “where to go” and doubts about “whether it was all worth it,” as another participant who experienced a high-risk flight journey described (35–39M). For many participants, starting life in Germany is much more complicated than expected: “The first months were sadness because it was all different from the utopia we had in our heads. […] Our dreams were shattered” (25–29F).

#### 3.2 Feelings of Loss of Agency, Status, and Being Undervalued

A perceived absence of progress in the structural integration process can also mix with a sense of injustice or lack of agency for refugees: “My life in Germany is imposed on me. If I can't establish anything for myself here, I will be more frustrated” (40–44F). Some perceive unforeseen bureaucratic restrictions as an affront to their sense of agency: “That they're not giving me a work permit or forcing me to do a particular job feels like imprisonment and that stresses me” (40–44F, different from previous). This feeling of a loss of control can erode an initial sense of motivation: “When I came to Berlin I had the plan to learn the language, and other plans to start my life here, it was a solid plan, but I couldn't do it. The Job Center did everything, I felt like I didn't have my own choice” (25–29M). Restrictions on movement were also mentioned as playing into a feeling of imprisonment and being thwarted.

Several participants described anxiety over a perceived loss of status due to not finding a next step suitable to their backgrounds. This prospect is potentially so unacceptable to some that it depletes their motivation. One participant spoke of friends and acquaintances who are unwilling to integrate because “they did a lot in their country of origin and now they have to start from ‘zero,’ and that is not ok for them” (25–29F). A fixation on the perceived loss of status can also be demoralizing: “I know a few people who always think about what was in Syria, what they studied, what […] and now they can't find their way in Germany. It's so hard” (30–34M).

Furthermore, not finding a purposeful activity and not feeling supported in the search for one are sometimes accompanied by a sense of not being valued by the host society. A young male who reported having “a lot of energy” and was eager for employment opportunities feels that his Job Center representative simply “forgets” about his case: “I don't feel really that in Germany people care about the young people who came with their huge potentials, care to guide them in the right direction” (25–29M). Some participants also feel undervalued during the structural integration process due to the “arbitrariness” of not being “allowed to do these things that others are” (30–34M), when they are restricted (in these cases: language course participation) and others are not: “I don't have any rights [because I don't have legal documents]. I was like a number, a file, and that's it. Not a person, but a file” (30–34M).

#### 3.3 Psychological Burden of Involuntary Inactivity

Several participants explained that the obstacles to building a life in Germany, such as lack of access to work and language courses, can lead to involuntary inactivity. Housing conditions were also emphasized as forcing inactivity: “Go to a camp, and you see how families live […]; they sit and watch TV all day, not because they want to” (35–39M). Inactivity, in turn, comes with deleterious mental health consequences, according to our participants. One participant stated that “most people get depressed” upon arrival in Germany because of the “very long waiting time to be able to do anything. This wait kills” (30–34F) and the concomitant loss of self-worth: “This feeling was continuous [before psychotherapeutic treatment]. A feeling that I'm not important [anymore].” Another participant reported: “I am depressed […] because I am sitting at home doing nothing” (40–44M). Several male participants described suffering from rumination because of a lack of activity and missing work as a distraction, “especially as someone who worked like a machine his whole life” (30–34M).

Showing that some refugees may have experienced the burden of lacking purposeful activity for extended periods before their arrival in the countries they settle in, one participant counted years of “sitting and doing nothing” (35–39M) in various countries on his way to Germany. “Feeling[s] of meaninglessness […] which are maybe bearable for a year” (30–34M) and anger about “life just pass[ing] by” (30–34M) result from months and years spent doing “absolutely nothing.” Even for those enrolled in language and integration courses, the waiting time until the next course begins poses a mental health challenge: “I had depression or something, doing a language course, waiting for the result, then a 2-month wait for the next course, doing nothing” (25–29M).

A pernicious added layer to these frustrations is a feeling of shame about receiving social benefits and the worry that their involuntary “sitting around” will feed into prejudices against refugees: “The people who don't like refugees, they say things like we are lazy and just sitting around our houses, but we are not” (30–34F). Some feel helpless in reacting to these judgments in light of how difficult they find building a life: “What can I do? People say: ‘Oh, he just wants to sit around.’ They don't see the reality” (30–34M). These feelings often co-exist with discomfort about receiving social benefits: “I don't like taking money from someone and then also have that be constantly be held against me” (40–44F), which many also attribute to not being familiar with social benefits as an institution from their home country.

Finally, it is of note that frustration over difficulties with structural integration and sitting around can lead to mental health problems, making it even harder to become active. One participant told us: “There's a fine line between you and giving up, as a refugee” (35–39M). Another participant (25–29M) suggested that there should be mental health check-ins at Job Center appointments to counteract this vicious cycle.

### Theme 4: Overwhelmed With Fundamental Tasks in the Integration Process

Fundamental daily tasks of integration include learning the host country language(s) (sub-theme 4.1.) and navigating the administrative processes (4.2.) involved not only in the asylum process but in everything from securing social benefits and housing, to getting certificates recognized and enrolling in courses, to seeking medical care. While many participants spoke about the stress of struggling with these tasks, they appear to have the potential to become so overwhelming that they impact mental health for some. This theme also includes examples of consequent withdrawal as well as mentions of poor mental health exacerbating everyday stressors.

#### 4.1 Language Learning

Almost all of our participants mentioned learning a new language as a primary stressor. For some participants, this stressor can take a significant psychological toll. In particular, refugees with limited educational backgrounds can experience learning German as severely distressing, especially those who are illiterate in their mother tongue: “I'm learning German and, in parallel, I'm trying to learn to write words in Persian […]. I think my psychological problems come from a pressure to learn. I think about it a lot” (age 40–44).

One participant criticized the German integration scheme for failing to “know the circumstances of the world” in sending people who have “never studied in [their] whole life” to standard integration and language courses (50–55F). Another said: “It is like a punishment to them, being sent to an integration course. […] Some of them had to visit a psychotherapist. So, imagine how much they suffered that they needed to visit a doctor for it” (40–44F). Mothers of young children reported stress due to lacking the time and space to study. Older age can also exacerbate the difficulties associated with a limited educational background: “And their ages range around the forties. Age plays a big role in language learning” (40–44F), and feelings of alienation can emerge from hopelessness about learning: “He [older, less educated acquaintance who is “in crisis”] doesn't understand a thing. He always feels estranged” (18–24M).

As addressed in Theme 1, pre-existing psychological issues can also be a reason for feeling overwhelmed with language learning. One participant (25–29M) felt unprepared to attend a language course because his “psychological status was not great” due to acute worries about his family and a stressful living situation in a housing facility. He demanded that these circumstances should be taken into account by Job Center and language program staff. The status quo, he feels, simply forces people to “go and fail.”

#### 4.2 Administrative Tasks and Bureaucratic Processes

Another facet of integration that was identified by almost all participants as a major stressor and by some as a cause of feeling psychologically overwhelmed was bureaucracy. The sheer number of bureaucratic processes [“Germany is the country of papers and bureaucracy. Always papers and appointments” (30–34F)], their incomprehensibility [“the language can't even be understood by Germans” (50–55F)], and the lack of assistance for foreigners [“structures and processes that do not exist in Afghanistan or Iran, […] and no one is there to advise you” (age 25–29)] are nearly ubiquitous sources of frustration.

For some participants, this stress from bureaucracy sounds as though it is of a severity that is pertinent to mental health: “[Bureaucracy] causes me tension in that a hundred ideas must be present in my head to perform 100 tasks every day” (30–34F), and as a consequence also to integration processes: “When I got that letter, I didn't understand anything in class all day because I just keep thinking about the letter. I had nothing but stress […]. This happens a lot” (40–44F). Everyday pressures also impede some refugees' efforts to overcome mental health struggles by pursuing meaningful activities: “Depression … I feel negative most of the time. I am trying to break through this negativity. […] Every morning after I get up, I tell myself, ‘today, I'm going to start something new’. But after you are faced with all these bureaucracies and pressures such as learning the language or not knowing what's going to happen tomorrow” (40–44F). One participant highlighted that bureaucratic demands can be particularly overwhelming immediately after arrival, when mental health is frail: “Refugees […] come from war and are very emotional, need a bit of motivation, but there is nothing but bureaucracy at the beginning, tac tac tac” (25–29M).

The stresses of bureaucracy sometimes interact with family dynamics. For example, one participant who struggles with a sense of overwhelm feels additional despair about not having achieved reunification with her husband because he was responsible for the family's administrative affairs in the past; this would “unburden” her. A few participants reported relying on their children to tackle bureaucracy because of their superior comprehension skills. One young participant (age 18–24) moved out of their family home because this responsibility became too stressful and all-consuming.

Feelings of being overwhelmed with bureaucratic processes can also arise from feeling mistreated and thwarted by administrative bodies. Several participants expressed finding them arbitrary and untrustworthy: “I have only heard lies from administrative bodies so far. They say one thing and do another. They use your statements […] against you” (40–44F). These negative experiences can have consequences for well-being, motivation, and integration: “It's even gotten to the point where, because of these problems [“they treat you as they wish”], I am less willing to make contact with people. This naysaying by administrative bodies makes me think, ok, then I guess nothing is possible, and I no longer make any effort at all. […] The poor treatment by authorities influences my thoughts and the rest so much that I let them out as anger toward my wife and my children. Or my wife says, ‘let's go somewhere,’ and I don't feel like it and say I have a headache” (30–34M).

### Theme 5: Social Disconnections With the Host Society and Fellow Refugee Communities

This theme captures various forms of social disconnection, showing links between the social aspects of integration and mental health. Regarding social integration with the host society, it covers experiences of xenophobia (sub-theme 5.1) and how participants experience an absence of close-knit family and other networks (5.2.), and thus, a lack of social support in Germany (5.3.), as well as particular risks from isolation in refugee camps after arrival (5.4.). This part of the theme captures instances of social disintegration negatively impacting mental health as well as this distress resulting in further withdrawal and demotivation.

Our understanding of social integration is not limited to examining the “bridges” between refugees and members of the host society (56). We also consider “bonds” within the refugee community and between migrant co-nationals to be vital parts of integration and a potentially significant source of support and solidarity. Therefore, this theme also addresses different forms of erosion of social cohesion among refugees and co-nationals, including stress and negativity from pre- and post-migration struggles, mistrust due to asylum status anxiety, and conflicts because of how some change their attitudes and behaviors in Germany (5.5.). Here, worries and mental health struggles, many related to integration, are shown to threaten social integration, potentially further jeopardizing well-being.

#### 5.1 Experiences of Xenophobia and Racism

While most participants characterized their reception in Germany as overall acceptable, even positive, or at least ambivalent, almost all participants reported experiences of xenophobia. This facet of exclusion and disintegration has the potential to act as a major stressor. According to our participants, slurs such as “Go back to your country!” (30–34M), “Why are you here?” (25–29M), and “Asylee!” (35–39F) from strangers in public spaces are not rare occurrences. One participant said that reading discriminatory headlines about refugees committing crimes makes him feel he does not want to go outside: “I cannot live well, I cannot walk on the street without thinking that others are looking: he's a refugee” (25–29M). Female participants perceive the hijab as a central source of discrimination: “Not everyone in Germany is racist, but the majority are, and I'm suffering from this, especially since I'm a woman who's wearing a hijab” (35–39F). One woman avoids public transport as a hijab-wearer for fear of “harassment from drunk people” (50–55F).

In personal encounters, our participants described facing false perceptions of themselves as “backward thinking, closed-minded extremists” (25–29M), “lower-level” (18–24M), and “barbaric” (25–29M) and always “having to prove yourself [as well-meaning]” (30–34F). Showing that discrimination is also experienced in interactions with actors involved in the integration process, one participant reported being in the midst of a discrimination complaint against the heads of her refugee housing facility for feeling looked down upon and ignored (25–29F). A German-language teacher supposedly told her students, “‘honestly speaking, I don't like Arab men”’ (40–44M).

On a political level, the rise of far-right, anti-immigrant sentiments in the German political landscape was mentioned as a concern by several participants: “There are AfD and NPD [far-right political parties] campaign posters that you see here that cause a deep-seated fear in migrants who can read German. This leads to stress and worries” (18–24M). Another said that being used as “pawns” in the political game between all parties “is really awful for us” (25–29M), a sentiment closely echoed by another participant who said that as a consequence of the treatment of refugees by the media, “we feel forced on people” (25–29M).

A few interviewees attributed almost all of their negative emotions and mental health struggles in Germany to feeling rejected and discriminated against. For example, a young woman (age 25–29) said that she “senses a hatred from the German people” and has “often been treated badly.” She described walking into her workplace in the mornings and having her greetings ignored by her German colleagues while observing that they do greet other Germans. Her predominant feelings in Germany have been “loneliness, hopelessness, isolation.” She talked about suffering from depression and feeling unable to engage in activities outside of work, connecting her poor mental health to the rejection she experiences: “The feeling that one doesn't belong here results in a loss of motivation, in being less active and in withdrawal.”

A young participant (age 18–24) who fled alone and attempted suicide in Germany said that they initially thought that “countries in Europe like Germany are safe places, where you can feel at peace.” They were shocked by what they found, having experienced several racist attacks, including a physical assault and an attack on the housing facility they lived in: “When I arrived, I realized that it's the opposite. Here there is racism; the lack of support is omnipresent. Everyone wants to succeed, but they put obstacles in your way.” This feeling of being discriminated against and unwanted had severe consequences for this individual's attitude toward integration, which they see as a process that has to be reciprocal: “I tried to integrate into this society, but they didn't want me to. […] If they don't want my integration into this society, then I don't want it either.” This participant said that “all of these difficulties” led them to attempt suicide because “someone who is not adult and in puberty is more easily hurt in their dignity,” emphasizing the vulnerability of very young refugees. They still do not feel safe: “The fear is deeply ingrained.”

#### 5.2 Perceived Lack of Close-Knit Social Networks in Germany

Another source of disconnection from the host country society presented in our interviews is a sense of alienation and loss regarding perceived differences in social life: “The social life I think here is very difficult, and I see this as the most difficult thing” (30–34M). Participants characterized their social networks in their countries of origin, to a great extent comprised of family, as being large (“I used to meet up to 150 family members per week”, 30–34M), close-knit (“safe, held-together units,” 25–29M), and involving frequent contact (“I spent most of my life, my whole time, in my friends' homes,” 30–34F). By contrast, many participants expressed feelings of alienation about how they perceive Germans' social lives: distant, cold, or even non-existent. Difficulty making social connections with Germans, a fear of adapting to this lifestyle, and feelings of isolation were reported as concomitant with these observations. Participants across genders, age groups, and countries of origin expressed these thoughts:

“I thought Germany was a highly-developed country and everybody was happy. But I don't feel people are happy here, especially the Germans. I'm afraid to become like them. […] German people lack a social life. […] They don't visit each other.” (Syrian, age 25–29).

“They are cold and take everything seriously, not like Eastern people who warm up quickly and make friends easily. […] Even friendships are cold.” (Afghan, age 18–24).

“I think this is a little bit scary [that she has not met neighbors of 2 years]; I feel like I am living alone.” (Syrian, age 30–34).

A young woman (age 18–24) attending school spent the first year in Germany hiding from her classmates during recess and “sat at home and did nothing” but watch television series in her free time because she was doing “terribly” emotionally from feeling ignored and rejected by her peers at school. She said she came to attribute this to cultural differences. “[In my country of origin], if you catch someone's eyes on the street randomly, you say ‘oh, hello!.’ In Germany, I think if I just smiled at someone randomly and said ‘hello,’ this person would think ‘piss off’.”

A Sudanese participant (age 35–39) saw the loss of social “nearness” as a tradeoff for a society in which the state assumes responsibility for meeting many needs that, in Sudan, would be within the purview of relatives, friends, and religious figures: “People in Sudan live together, help one another, just do everything for one another. [This is something] I miss very, very much.”

#### 5.3 Lack of Social Support and Feelings of Community

These perceived differences in social life lead to a sense of loss of emotional support for some participants, affecting their mental well-being. One woman feels “exhausted” (30–34F) as a consequence of not being in the type of “social environment that gave [her] comfort.” Another participant said that in the close-knit community in the country of origin, he “was not afraid of the future or anything” (25–29M). He described his current state in Germany, on the other hand, as being marked by depression, anxiety about building a life, and feeling alone with his problems. Another participant similarly feels that he is “not allowed to fall” in Germany because, unlike back home, no one will catch him: “I have to be so strict I cannot fail, and just thinking about it is very stressful” (25–29M).

Seeking long-distance social support from the familiar network is not always an option due to a reluctance to burden family and friends who are already perpetually worried. For example, a young man (25–29M) describes that he “would love to share that [he] feel[s] tired and stressed,” with his family, but refrains so as not to worry them. When he is feeling particularly low, he avoids video calls or “put[s] on a mask.”

#### 5.4 Isolation in Refugee Camps

A few of our participants described the temporary residence in refugee mass accommodation after arrival in Germany as a period of social isolation in an already difficult time [“I always wonder if Germany is aware how depressive the people are that they are putting in mass accommodation” (25–29M)] with severe consequences for mental health and integration:

“The way they are isolating refugees in camps is totally destroying them. After all the suffering those refugees had to go through to reach Germany. […] At the time they left the camps, they are already let down. I had friends who were so motivated when they first arrived in Germany. But they were isolated in camps for about 6 months until they got the residency. They were totally devastated by then. It took them a while to regain their mental health and be able to start again. But unfortunately, not all of them were capable of getting over it” (30–34F).

According to another participant (25–29M), the isolation in mass accommodation also means that although “there are many good organizations [promoting refugee social integration] […],” it is difficult to become aware of these programs whilst living there: “I didn't see them for 2 years. […] Events with others, with Germans, there weren't any. Or too few, and you have to find them yourself.” Due to the psychological fatigue from flight and poor living conditions in mass accommodation, seeking out events is nearly impossible, according to this participant: “if you're in a camp, you have no motivation, zero motivation. […]. The beginning is very difficult.” He emphasized the importance of social connection in the initial phase of integration: “Maybe a word [from the host society] would help more than money and an apartment at the beginning,” and argued that given the mental health risk of those in housing facilities, mental health care should be integrated or accessible on site: “I am surprised how there are no psychological support teams to work in the housing facilities […] in an advanced country like in Germany […], but with refugees, it seems like they don't care about our psychological issues.”

Restrictions on visitors in some housing facilities and security measures also make several participants feel isolated: “we have to show our card like we are in jail” (30–34M).

#### 5.5 Lack of Social Cohesion Within Refugee and Migrant Communities

Participants also experience rifts with co-nationals and fellow refugees in Germany for various reasons.

“There are also divisions between Farsi-speaking people. They do not stand by one another” (age 25–29).

“I have not interacted much with any Arabs. Unfortunately, with all due respect, there were some fights between the Arabs I met [here] and me. I could not cope with the Arabs” (age 30–34).

A few of our participants reported an inability to turn to people from their own community for connection and support because “most of them have their own troubles and prefer to be left alone” (30–34M) or because “they are not psychologically stable, always thinking […], the Syrians in Germany are not like the ones in Syria” (age 25–29). Some have “deliberately moved away from [Arabic friends]” to escape the “negativity” and “discouragement” that apparently prevails in some Arabic refugee communities due to past and ongoing stressors: “In the camps for example, […] they say negative things, there are obvious problems these people have experienced, so the conversations always turn into the negative” (age 30–34).

Within refugee housing facilities, stress-inducing conversations and gossip about the asylum process can be the cause of a psychologically damaging atmosphere: “[…] in the camp […], people were talking about the trial and who got rejected or accepted! It was so stressful to witness all of this […]” (45–49M). Additionally, refugees appear to experience highly dysfunctional social environments due to crowding in these large, temporary housing facilities: “We were in mass accommodation for a year and 6 months, meaning 70 people in a gym—the conditions were terrible. Police were there every day […]. There were drugs, fights between residents, everything” (age 30–34).

Another potential threat to social cohesion, and thus, a threat to social support within refugee communities, appears to be mistrust and suspicion of others' intentions and grounds for seeking asylum, which often arises out of comparisons: “There are people I know very well who had no problems in [country of origin] and were nevertheless granted asylum. […] They just stay at home and get social benefits, while [we] try with all our strength to achieve something […] There has been confusion between those who deserve asylum and those who do not deserve it” (age 30–34). These statements often arise in the context of a participant reasoning that their efforts should be but are not rewarded with greater security than less engaged refugees receive: “The migrants who only eat and sleep, they could be treated differently” (18–24M). Frustration about the perceived lack of influence over one's fate may play into these perceptions.

Another participant (30–34M) who was “shocked” that their application for refugee status was rejected even considers some whose applications were accepted but “who don't deserve asylum” as a potential threat, as “dormant cells of the regime” who “carry news and reports about refugees living in Germany to the [country of origin] regime.” This transfer of the suspicion bred by political persecution in the country of origin to German refugee communities was framed as an obstacle to engaging within these communities by another participant: “Until this moment, I still check around me every time I speak to see if anyone has heard or not. Sometimes when I attend a lecture about Syria, I get the feeling that someone is monitoring me” (age 18–24). Overall, these striking instances of mistrust and comparison, while not connected to mental health directly by our participants, may contribute to feelings of rejection and isolation.

Finally, several clashes arise within the refugee community as a result of behavioral adaptation processes that cause distress. An LGBT participant (age 30–34) who feels free to express their identity in Germany experiences distressing bullying in a refugee housing facility. Several of our female participants reported feeling stressed by clashes between their lifestyles in Germany and certain community members' values: “I [live] alone. I get a lot of criticism because of that from [my] community, […] these criticisms put a lot of pressure on me” (18–24F). Some older participants reported feeling distressed by the lack of cultural cohesion amongst co-nationals in Germany. For example, one participant (50–55F) said that seeing young people from her country of origin “considering [themselves] German” and “not greeting her” in German class affects her ability to learn the language: “If I am not comfortable, I cannot learn.”

These examples show that stress from pre- and post-migration adversities may have an indirect deleterious effect on mental health by eroding certain sources of social support. As a consequence of these multiple disconnections from Germans and co-nationals and fellow refugees alike, one participant feels left without a home: “I am distant from [both]. I have become very isolated” (25–29F).

## Discussion

Our study identified five themes capturing different links between mental health problems or significant negative emotional experiences and integration processes as prioritized by refugees recently resettled in Germany. The scope and content of our study provide a comprehensive overview, touching on all domains and facets of integration that were important to the participants. It is of note that all our themes were manifest among participants from different age groups, genders, cultural backgrounds, and from three different German urban areas.

Our first theme addresses how lasting distress from past adverse experiences as well as ongoing worries about those left behind in the homeland can seriously impede refugees' ability to pursue activities key to integration. Specifically, several participants expressed a sense of being hindered by a “head full of knots,” a shortage of “brain capacity” or “being stuck,” “unable to overcome the grief,” “obsessed” with checking on those left behind and “unable to do” things like learning a new language or “unable to do anything” at all. While policy analyses have noted the potentially deleterious effects of mental health problems from adverse experiences on integration in their considerations [e.g., (57)] and some quantitative studies have found these correlations (29), our participants' reports add personal accounts of these effects. One participant's demand for psychotherapy as a prerequisite for integration shows that some refugees interpret their own situations as characterized by functional impairment hindering successful integration.

The second theme addresses how prolonged uncertainty in the asylum process and even afterward, when statuses are still limited to a few years at a time, has caused many of our participants substantial distress. They reported fear, anxiety, fatigue, and feelings of being at the mercy of a process they cannot influence—feelings of loss of control being a potential primary source of post-migration stress among refugees (58). These experiential reports add details to our understanding of the association between legal status insecurity and refugee mental well-being (16, 20, 24). Our participants also described that the burden of this uncertainty, like past adverse experiences, can lead to deactivation and that the threat of being sent away erodes motivation to participate and sense of belonging. It appears that legal status insecurity elicits feelings of being rejected or not valued by the host society and doubts about whether any steps forward in host society are worthwhile.

Our third theme includes accounts from participants who suffer from feeling stuck and thwarted in various ways in their attempts at “starting a new life,” especially on the level of joining the labor market in a job appropriate to their background or taking preparatory steps like completing language courses. Unsurprisingly, those who had made substantial progress in their education or in their career before flight and are not close to the end of their careers were most anxious about finding meaningful and suitable activities. They reported experiencing “depression” because their efforts to advance their lives are perceived as fruitless. The loss of direction in life can be “tiring,” and some participants have felt a burdensome lack of direction throughout their entire flight and post-flight life. Feelings of loss of agency and status and of not being valued also plague many of our participants.

The involuntary inactivity that follows from struggling to start life was described by participants as threatening to their mental health. They said it “kills” psychologically, brings on “unbearable meaninglessness” and feelings of no longer “being important” and “life just passing by,” which mix with shame over receiving social benefits. Like male interviewees in a refugee camp study in Turkey (36), who reported feeling “bored and offended” because of not being able to work, some of our male participants feel forced into an unfamiliar and pride-eroding situation. In line with another German interview study, we found that the conditions in refugee housing facilities are often described as contributing to inactivity (37). These feelings of powerlessness, meaninglessness, lack of control over the future and passivity, as well as their mental health ramifications, have been described previously, for example, in a study titled “A Life in Waiting” (59) on refugees stuck in transit in Greece. It is striking that many of our participants, who have been living in a country they intend to stay in for at least several years, still feel stuck in waiting. Previous explorations of the role of active participation in fostering self-esteem, self-worth, a sense of purpose, and an alleviation of mental health problems among refugees [e.g., (60)] complement our findings in this theme.

The fourth theme presents the psychological toll of feeling overwhelmed by fundamental tasks in the integration process, namely, language learning and bureaucratic processes. Language learning struggles come with “psychological problems” like “pressure,” “feelings of punishment,” and “estrangement,” especially for those with pre-existing mental health problems and those with limited educational backgrounds, a challenge that has been previously addressed (61, 62). While refugees' struggle with Germany's bureaucracy has been reported elsewhere (63), the psychological toll of bureaucratic hurdles on refugees appears to be rarely discussed in the literature. However, another German interview study also found that the lack of knowledge about processes and unpredictable or unclear administrative demands result in helplessness and loss of self-esteem (37). Overall, female participants expressed a sense of feeling overwhelmed more often than male participants, whose stress about bureaucratic processes tended to manifest in anger about perceived mistreatment and restrictions.

Finally, in the fifth theme, we identified several forms of social disconnection that were linked to mental distress by our participants explicitly or interpreted by us as threatening to well-being under the assumption that social support and social embeddedness are crucial to it (64). Experiences of xenophobia and racism were reported by most participants, consistent with previous findings (65). The link between experiences of xenophobia and racism and refugee mental health has been previously evidenced in the literature [e.g., (26, 66)]. While many of our participants only felt somewhat impacted, others reported strong feelings of rejection and not belonging, loss of dignity, sometimes fear, and the urge to withdraw socially and give up on integration. Our interviewees were also aware of and distressed by the rise of anti-immigrant sentiments in Germany and described feeling instrumentalized in political debates in a way that harms their relationship to host society communities.

Beyond rejection, our participants described experiencing a clash between Germany's forms of togetherness, which they see as “cold,” “distant,” or even absent, and the close-knit communities they come from. Some participants reflected on this as the clash between collectivist and individualist cultures (67). Several reported a lack of social support in the absence of their familiar social environment and feelings of pressure or exhaustion from living without their social safety net. While the impact of missing social support on refugee mental health has been discussed [e.g., (12)], our results suggest that it would be interesting to explore further whether there are certain forms of social support, not just social support *per se*, that are missing. In the early stages after arrival, complete isolation from the outside world in reception centers is a major threat to well-being, as others have reported [e.g., (37)]. Our participants offered striking warnings about the potential long-term harms of isolation and restrictive, stressful, even “inhumane” living environments at a time of severe vulnerability.

Our participants also reported rifts with fellow refugees and other co-nationals living in Germany. These represent threats to integration when integration is seen as consisting of both bridges between migrant and non-migrant communities and bonds within migrant communities (56). These conflicts appear to stem in part from flight and migration-related mental health problems, presenting another instance of mental health influencing integration. Our participants reported pervasive negativity among refugee communities because members of the community are “not psychologically stable.” Pervasive stress and talk about legal status matters, including unfavorable comparisons with those “who don't deserve asylum,” further damage social cohesion. While migration's effects on social cohesion, in general, have been discussed in the literature [e.g., (68)], social cohesion within refugee communities has rarely been addressed. One existing study on refugee activism found that legal status hierarchies cause rifts in refugee movements (69). Some individuals also experience stress within their community due to how they break with expectations in their new environment, a form of acculturation stress described in the migration literature (70). We argue that these erosions of solidarity pose a threat to mental health as well.

In reflecting on our results, it becomes clear that there is ample potential for interconnections between the mechanisms described within different themes. One form of connection between the themes emerges from the bidirectionality of effects. If mental health problems and feelings of uncertainty, rejection, or frustration can impede integration, and reduced progress with integration can cause or exacerbate mental health problems, then the potential for vicious cycles is evident. Secondly, the dynamics described in our themes could multiply one another because of the close connection between domains of integration, for example, between labor market integration, social networks, and language (71).

### Implications for Concepts and Policymaking

Our study supports Ager and Strang's (2) understanding of health as a “means and marker” of integration in the sense that it is both “an important resource for active engagement in a new society” and an outcome of successful integration policy. However, Ager and Strang limit their understanding of health as an outcome of successful integration policy to a demand for adequate healthcare as a part of integration measures. Our analysis supports health as an indicator of successful integration in a much broader sense: various domains of integration and their interplay have the potential to strengthen or erode refugee mental health and well-being. The close relationship between living conditions and mental health is not unique to refugees, and neither is the resulting public health imperative of providing living circumstances that foster mental health [e.g., (72–74)]. The WHO's “Health in All Policies” approach (75) encapsulates this demand. However, as others have previously argued, “Health in All Policies” is particularly relevant in the migrant and refugee context (8, 76). In a population that faces uniquely severe threats to well-being, “mental health” should not be conceived and treated in the medicalizing, individualizing sense, but as a direct distress response to adverse circumstances (77, 78). The term “refugee mental health” thus represents mental health as inextricably linked to the circumstances faced by this population pre-, peri-, and post-migration (79), both as a direct outcome of adequate conditions and as an important resource for integration.

It thus follows that integration policy is also health policy and vice versa. In Germany, refugee mental health care could be improved by ensuring immediate full access (80), more screenings and checkpoints in, e.g., refugee housing facilities and Job Centers, as suggested by one of our participants, and the development of lower threshold psychosocial interventions and community-based approaches as a way of meeting demand and connecting mental health needs to broader needs (81–83).

On the integration policy side, our study demonstrates the need for quick but high-quality, reliable asylum procedures (44) and the need to reconsider whether the legal status hierarchy is justifiable given its deleterious impacts (84). Ensuring immediate complete access to institutions and opportunities such as permission to stay for full vocational training to all new arrivals could be beneficial in myriad ways (57), if not for long-term integration, then for the sake of international development (84). The introduction of professional mentoring programs, such as those under development in Austria, Norway, and Switzerland (57), and easing access to the labor market by replacing certificates with skills tests and opportunities to learn on the job (85) could promote participation. Housing conditions need to be compatible with an active life (85). The diversification of integration routes is also important: for example, the diversification of language courses according to background and goals (57). Furthermore, a streamlining, shortening and simplification of laws and processes is needed (85), both to benefit refugees lost in a bureaucratic jungle and for organizations working with them (84). Finally, the facilitation of community projects that are easy to access has the potential to address multiple obstacles that our participants describe and simultaneously foster social connection and cohesion (81, 86).

### Limitations

Potential selection biases in participant recruitment represent an important limitation in our study. While we achieved our goal of recruiting some participants who are hard to reach, such as older and illiterate refugees, there are still undoubtedly self-selectivity mechanisms involved. Very highly educated participants were clearly more likely to self-select into our sample, as the high proportion of university-educated interviewees shows. All participants were able to follow through with an interview appointment, and they were willing to open up. They might have been particularly keen to voice their perceptions of what is not working in their integration efforts. Our a priori focus on challenges and problems in the present study may also have skewed the overall impression of refugees' experiences to the negative—a further limitation. A sister study on resilience based on the same data offers another perspective (49).

Furthermore, it was not the aim of this study to diagnose mental health problems. Thus, the instances of poor mental health identified cover a broad range. It is a strength of our research that we were able to offer participants interviews in their preferred language and with culturally competent interviewers. However, despite a quality check, translated transcripts may not be linguistically precise and do not reflect subtleties in tone. The necessity for multiple interviewers also added complexity to the study and may have introduced some systematic differences between interviews. The different cultural backgrounds of researchers, participants, and interviewers may, of course, also have resulted in some misunderstandings, particularly with regard to questions and concepts relating to mental health. This study did not capture the experiences of refugees living in rural settings in Germany.

## Conclusion

This study examines the complex and intertwined relationship between mental health and integration for a diverse sample of recently-arrived refugee adults in three different urban areas in Germany. Our findings shed light on various ways in which, on the one hand, poor mental health negatively impacts the ability to pursue integration, and, on the other hand, difficulties integrating within different domains contribute to mental health problems. This study has policy implications for stakeholders interested in integrating refugee populations across Germany, including the need to ensure mental health service provision, improve the speed and quality of the asylum-seeking process and reevaluate the legal status hierarchy, provide integration and language courses that are sensitive to individual differences, including mental health status, reduce bureaucratic demands, improve housing conditions, increase awareness regarding the impact of discrimination from the host community on the integration of incoming populations, and support initiatives that combat isolation and disconnection. Innovative solutions to challenges identified by members of the refugee community in Germany stand to benefit mental health and integration outcomes simultaneously.

## Data Availability Statement

The datasets presented in this article are not readily available because the data consists of complete semi-structured interviews that reveal a lot of personal information, so we would not be able to safeguard the anonymity of our participants by sharing the data, even if no explicit identifications were included. Requests to access the datasets should be directed to lena.walther@charite.de.

## Ethics Statement

The studies involving human participants were reviewed and approved by Ethics Commission of the Charité–Universitätsmedizin Berlin. The patients/participants provided their written informed consent to participate in this study.

## Author Contributions

LW, TT, EH, and MB conceived of the study. LW developed the mental health part of topic guide. LW, DR, JA, and further collaborators collected the data. LW and JA coded the transcripts. LW performed the thematic analysis with feedback and input from JA. LW wrote the manuscript. DR gave feedback and input on multiple drafts of the manuscript. UF, TT, EH, and MB critically reviewed and made edits to the manuscript. All authors contributed to the article and approved the submitted version.

## Funding

This work was supported by the German Federal Ministry of Education and Research (grant number 01UM1812BY). DR was supported by the U.S. Fulbright Scholar Program. We also acknowledge support from the German Research Foundation (DFG) and the Open Access Publication Funds of Charité - Universitätsmedizin Berlin.

## Conflict of Interest

The authors declare that the research was conducted in the absence of any commercial or financial relationships that could be construed as a potential conflict of interest. The handling editor declared a shared affiliation with the authors at time of review.

## Publisher's Note

All claims expressed in this article are solely those of the authors and do not necessarily represent those of their affiliated organizations, or those of the publisher, the editors and the reviewers. Any product that may be evaluated in this article, or claim that may be made by its manufacturer, is not guaranteed or endorsed by the publisher.
